# Frequency of Unrecognized and Undertreated Dementia Risk Factors in Community‐dwelling Latino and Black Americans

**DOI:** 10.1002/alz70860_107565

**Published:** 2025-12-23

**Authors:** Christian Lachner, Yoav D Piura, Paula Aduen, Leah Schecter, Minerva M. Carrasquillo, Richard O White, Neill R. Graff‐Radford, Gregory S Day

**Affiliations:** ^1^ Mayo Clinic in Florida, Jacksonville, FL, USA; ^2^ Mayo Clinic, Jacksonville, FL, USA

## Abstract

**Background:**

Disparities in structural and social determinants of health in Latino and Black communities may increase the burden of Alzheimer disease and related dementias (ADRD). Identifying dementia risk factors, which may account for up to 45% of ADRD risk, is crucial to implement effective risk‐reduction strategies. This study aimed to assess ADRD risk factors in community‐dwelling, cognitively normal, Latino and Black Americans by measuring the frequency of modifiable dementia risk factors through subjective and objective measures.

**Method:**

Community‐dwelling, cognitively normal, Latino and Black adults (≥45‐years‐old) were enrolled in a longitudinal study of memory and aging at Mayo Clinic in Jacksonville, Florida. Baseline assessments conducted between Mach 2022 and November 2024 included demographics, self‐reported race/ethnicity, and history of dementia risk factors. Socioeconomic deprivation [Area Deprivation Index (ADI) State scores] and modifiable dementia risk factors were reported by participants and directly measured, including hypertension (BP >130/80 mmHg), obesity (BMI≥30), hyperlipidemia (LDL>100 mg/dL), hyperglycemia (HbA1C >5.6%), low vitamin B12 (B12<350 pg/mL), hearing loss (bedside assessment), vision loss (bedside assessment), and depression (Geriatric Depression Scale ≥5).

**Result:**

105 participants (47 Latino, 55 Black), 64.7 (±8.8) years‐old, female 66 (63%), with 16 (±2.3) years of education were assessed. Black participants experienced higher socioeconomic deprivation than Latino participants (median ADI 7 vs 3; *p* <0.001). Dementia risk factors were common in Latino and Black participants. These included, hypertension (55.3%, 70.3%; *p* = 0.291), hyperlipidemia (58.7%, 55.8%; *p* = 0.908), obesity (29.8%, 45.5%; *p* = 0.263), hyperglycemia (51.1%, 49.1%; *p* = 0.834), low vitamin B12 (23.9%, 28.0%; *p* = 0.270), hearing loss (19.1%, 7.3%; *p* = 0.111), vision loss (85.1%, 85.2%; *p* = 0.771), and depression (6.4%, 5.5%; *p* = 0.168). Objective measures revealed a higher frequency of risk factors compared with participant‐provided history, highlighting a substantial proportion of unrecognized or inadequately treated risk factors: hypertension (62.3%, 45.1%; *p* = 0.067), hyperlipidemia (57.6%, 47.5%; *p* = 0.004), hyperglycemia (49%, 20%; *p* = <0.001), vitamin B12 deficiency (27.8%, 9.3%; *p* = 0.170), and depression (6.9%, 11.9%; *p* = 0.009).

**Conclusion:**

Modifiable dementia risk factors were detected in most cognitively normal Latino and Black adults engaged in a longitudinal study of memory and aging. Objective measures highlighted unrecognized and undertreated risk factors. Active surveillance programs are needed to detect and treat modifiable ADRD risk factors in underrepresented populations.

